# Developmental origin of tendon diversity in *Drosophila melanogaster*

**DOI:** 10.3389/fphys.2023.1176148

**Published:** 2023-04-18

**Authors:** Blandine Moucaud, Elodie Prince, Krzysztof Jagla, Cédric Soler

**Affiliations:** GReD Institut, UMR CNRS 6293, INSERM U1103, University of Clermont-Auvergne, Clermont-Ferrand, France

**Keywords:** tendon, *Drosophila*, muscle, development, myogenesis

## Abstract

Myogenesis is a developmental process that is largely conserved in both *Drosophila* and higher organisms. Consequently, the fruit fly is an excellent *in vivo* model for identifying the genes and mechanisms involved in muscle development. Moreover, there is growing evidence indicating that specific conserved genes and signaling pathways govern the formation of tissues that connect the muscles to the skeleton. In this review, we present an overview of the different stages of tendon development, from the specification of tendon progenitors to the assembly of a stable myotendinous junction across three different myogenic contexts in *Drosophila*: larval, flight and leg muscle development. We underline the different aspects of tendon cell specification and differentiation in embryo and during metamorphosis that result into tendon morphological and functional diversity.

## 1 Introduction

The musculoskeletal system comprises an assembly of distinct tissues. Among these tissues, the tendons ensure that the correct transmission of muscle contraction force is applied to the skeleton. Although *Drosophila melanogaster* is an established model used to better understand the cellular and molecular events in the conserved myogenesis process (Deng et al., 2017; Laurichesse and Soler, 2020; Boukhatmi, 2021; Luis and Schnorrer, 2021; Junion and Jagla, 2022; Rout et al., 2022), some studies have also highlighted common features in the development of muscle attachment sites between vertebrates and invertebrates (Schnorrer and Dickson, 2004; Schweitzer et al., 2010; Hasson et al., 2017; Valdivia et al., 2017).

Since flies lack an internal skeleton, their muscles are connected to the exoskeleton (cuticle) with specialized tendon-like cells (also called apodemes). Both tendon-like cells and muscles, but also other tissues such as fat body, secrete extracellular matrix proteins that form the equivalent of a Myo-Tendinous Junction (MTJ) present in vertebrates. As a holometabolous flying insect, *Drosophila* develop specialized muscles to enable locomotion, first as a crawling larva and later as a walking and flying insect. Larval somatic muscles form a segmentally repeated pattern of thirty multinucleated myofibers, whereas adult flies exhibit muscles with wider range functions such as direct and indirect flight muscles, leg muscles and muscles that facilitate jumping (Bate, 1993). Accordingly, *Drosophila* possess a variety of morphologically distinct tendons. In larvae, the extremities of each monofiber are anchored to a single tendon cell (Volk and VijayRaghavan, 1994; Frommer et al., 1996). The powerful indirect flight muscles are composed of multiple fibers that are connected to the exoskeleton through an array of tendon cells (Fernandes et al., 1996) and tendon cells in the leg form long internal tubes around which muscle fibers are arranged (Miller, 1950; Soler et al., 2004)**.** Whereas the initial cell specification of each tendon type relies on the induction of the Stripe (Sr)/Egr-like transcription factor (Fernandes et al., 1996; Frommer et al., 1996; Vorbrüggen and Jäckle, 1997; Ghazi et al., 2003; Soler et al., 2004)**,** subsequent steps in the genetic program must further distinguish tendons to achieve their specific terminal differentiation. As such, there is an opportunity to identify the mechanisms that lead to the formation of differentiated tendons that are adapted to specific muscles.

Here we review existing knowledge on the development of tendons that connect with larval and insect flight and leg muscles, in addition to recent findings that emphasize reciprocal interactions between developing tendons and muscles.

## 2 Signaling regulators of tendon cell specification

In *Drosophila*, all tendon cells are characterized by the expression of *stripe* (*sr*), which is the earliest known marker of tendon cell specification (Frommer et al., 1996). *sr* encodes a triple zinc-finger transcription factor and is a member of the early growth response family, whose vertebrate orthologous EGR1 and EGR2 are also involved in regulating tendon development (Lejard et al., 2011). *sr* encodes for two major mRNA transcripts, a short isoform *srB* that differs from a long isoform *srA* in its absence of exon1 (Lee et al., 1995; Frommer et al., 1996). Across muscle systems, different combinations of inductive and repressive signals generate complex temporal and spatial patterns of *sr* expression.

### 2.1 Larval muscle attachment sites

The formation of a stereotypical larval muscle pattern relies on correct location of Sr+ cells in the epidermis (Volk and VijayRaghavan, 1994; Frommer et al., 1996; Becker et al., 1997; Volk, 1999). Indeed, tendon-like cells provide positional information that controls the direction of myotube migration. Thus, in cases of mutant embryos, in which tendon-like cells are missing or have failed to differentiate, the muscles are severely disorganized (Volk and VijayRaghavan, 1994). Moreover, ectopic expression of *sr* leads to the specification and differentiation of newly formed muscle attachment sites (MAS) within the epidermis that can attract muscle fibers (Frommer et al., 1996; Becker et al., 1997).

**FIGURE 1:**
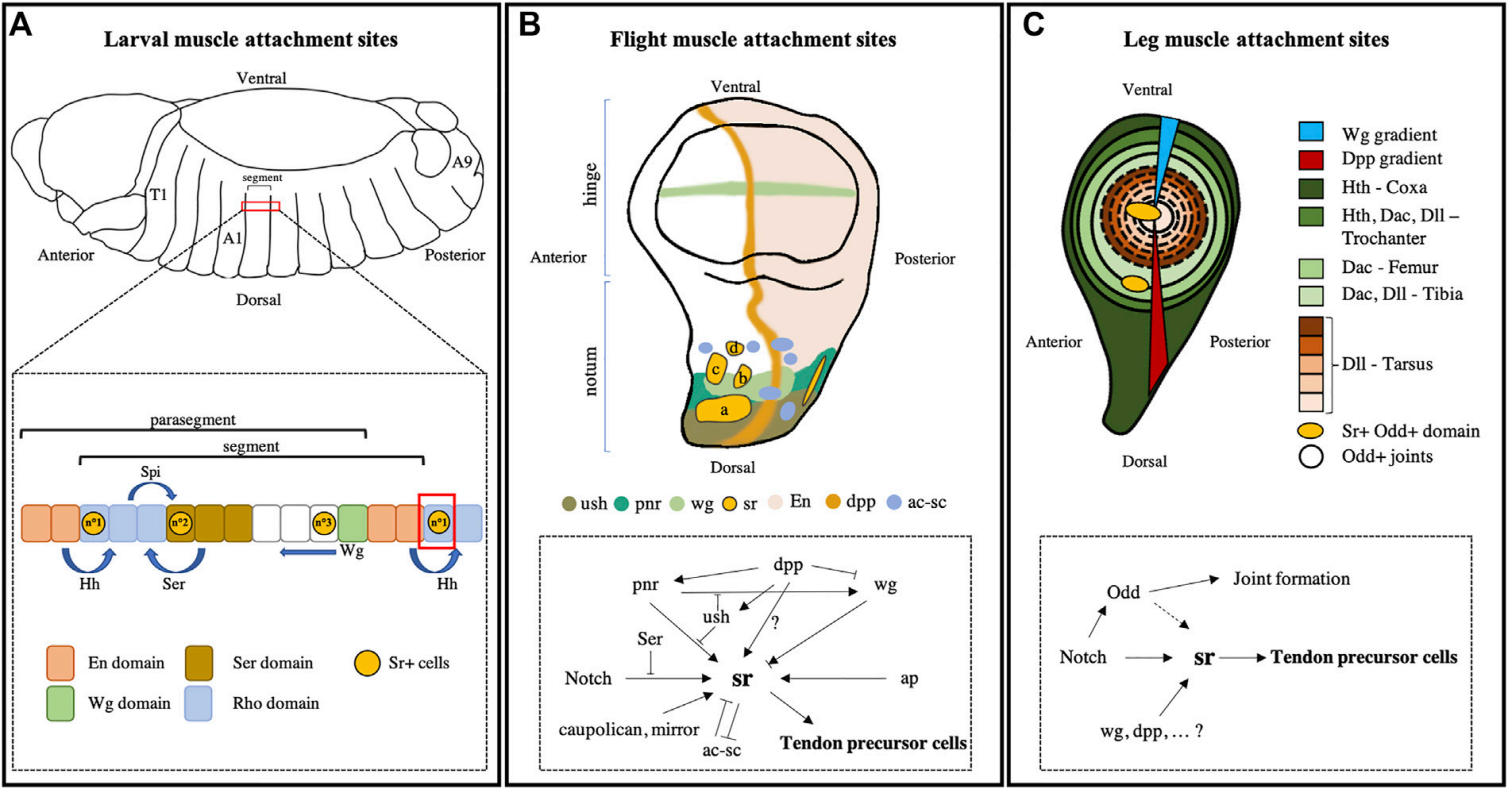
Signaling regulators of tendon cell specification. **(A)** During embryogenesis, the *Drosophila* embryo is divided into 14 parasegments (PS) and then further divided into 15 segments. Each PS is delimited by the Engrailed (en) and Wg domains that secrete Hh and Wg signaling molecules, respectively. These signals delimit segments that comprise 12 rows of cells and can be further subdivided into different domains of expression (Rho+, Ser+, Wg+, and En+ domains). In the three rows of each segment, *sr* expression is induced through the differential combination of patterning signals. *sr* expression in row n°1 is induced by Hh signaling in a Rho+ domain. In row n°2, *sr* expression is under the control of Spi/EGFR signaling in a Ser+ domain. Lastly, a third Sr+ row is defined by the Wg signaling pathway. **(B)** In the *Drosophila* wing imaginal discs, *sr* is expressed in five clusters located in the notum: Three in the anterior region (a–c) that give rise to the anterior attachment sites of the so-called Dorso-Longitudinal flight Muscles (DLM), one in the posterior region at the origin of the posterior attachment sites of these same DLM and one (d) that corresponds to the future attachment site of the TDT or jump muscle (Ghazi et al., 2003). The initiation of *sr* expression is mediated by several regulatory interactions and is dependent on local signaling factors that ensure the segmental subdivision of disc territories. The expression pattern of these genes shows that *sr* expression is regulated differently in each cluster. Dpp demarcates the antero-posterior axis and positively controls *sr* expression through pnr and by limiting Wg activity. N is required for the induction of *sr* expression in all clusters, however its activity is antagonized by its ligand Ser. At high levels, Wg suppresses *sr* expression, whereas a moderate level of Wg initiates *sr* expression. Apterous (ap) and Iroquois family gene products (*caupolican* and *mirror*) positively regulate *sr* expression. Sr and ac-sc exhibit mutually antagonistic activities. Arrows represent gene activation and arrow bars indicate repression (adapted from Ghazi et al., 2003). **(C)** In leg imaginal discs, Wg and Dpp morphogen diffusion create a gradient from the ventral to the dorsal region establishing the first pattern of the dorso-ventral axis. Then, this gradient induces the expression of segmental genes (*Hth, Dac*, and *Dll*) along the proximo-distal axis. The Notch pathway defines boundaries between the presumptive segments by inducing *odd-skipped* family gene expression in a ring of epithelial cells. Notch is also required to trigger *sr* expression in discrete domains along the odd-positive rings. This way, a total of seven Sr-positive clusters are specified (only two are represented here), which give rise to long internal tendons.

The activation of *sr* expression in embryos is controlled by segmental polarity signals, especially through Hedgehog (Hh) and Wingless (Wg) signaling pathways (Piepenburg et al., 2000; Hatini and DiNardo, 2001). Expression of these signals delimits transitory boundaries, called parasegments (PS) (Figure 1A). PS are characterized by the Engrailed (En) and Wg expressing cells that secrete Hh and Wg signaling molecules, respectively. The Hh signal binds to its receptor, called Patched (Ptc), expressed at the surface of adjacent cells leading to the activation of transcription factor Cubitus interruptus (Ci) (Alexandre et al., 1996; Von Ohlen et al., 1997). Wg is a member of the Wnt family and binds to its receptor Frizzled (fz). In the Wnt/β-Cat canonical pathway, Fz activation leads to the accumulation of β-Cat, which translocates to the nucleus and heterodimerizes with the transcription factor pangolin (dTCF) to activate target gene transcription (Bejsovec, 2018). In each PS, the Hh secretion generates an anterior to posterior gradient, whereas the posterior to anterior Wg gradient is established from the next posterior boundary (Sanson et al., 1999). Opposing gradients of the Hh and Wg signals demarcate a Serrate (Ser) positive domain in each PS (Gritzan et al., 1999). Then, Hh and Ser signals delimit a Rhomboïd (Rho) positive domain in the anterior part of the PS (Gritzan et al., 1999). Ser is a Notch ligand and rho regulates the synthesis of the EGFR (Epithelial Growth Factor Receptor) ligand, Spitz (Spi). Thus, each segment is composed of 12 cell rows, of which three express *sr*. In the most anterior row (n°1), *sr* expression is induced through Ci-mediated Hh signaling in the Rho+ domain (Piepenburg et al., 2000; Hatini and DiNardo, 2001). In a median position, a second row of *sr* expression is induced under the control of Spi/EGFR signaling in the Ser+ domain. Lastly, in a more posterior domain, a third Sr+ row is defined by Wnt signaling pathway (Hatini and DiNardo, 2001) (Figure 1A). Alterations to any of these signaling pathways may result in a loss of *sr* expression in the corresponding row. For instance, in Hh mutant embryos, *sr* expression is missing in rows n°1 and n°2, as *sr* expression in row n°1 is controlled by ci and in row n°2 *sr* expression is controlled by EGFR, which itself depends on the Hh signaling pathway (Piepenburg et al., 2000). Thus, the initial determination of tendon-like cells in an embryo requires signaling positional information within the ectoderm but appears independent of muscle presence, as shown by the expression of *sr* in *twist* mutant embryos that lack mesoderm (Becker et al., 1997). However, continuous expression of *sr* is maintained only in MAS that are subsequently connected to muscles, indicating that a signal from the attracted muscle affects the regulation of *sr* expression (Becker et al., 1997). As described above, *sr* gene encodes for a short *srA* and a long *srB* isoforms (Lee et al., 1995; Frommer et al., 1996). *In situ* hybridization showed that *srB* is first expressed at the early phase of tendon cell determination, whereas *srA* isoform is upregulated at a later stage (Volohonsky et al., 2007) and contributes to the terminal differentiation of tendon cells (see further details described below).

### 2.2 Induction of *stripe* expression in muscle attachment sites of adult muscles

Adult flight and leg muscles develop from a pool of adult muscle precursors (AMP) that are present on the surface of the wing and leg discs, respectively (Gunage et al., 2017; Laurichesse and Soler, 2020). Wing and leg discs are epithelial precursors to adult wings and legs as well as most of the thoracic body wall (Cohen et al., 1993). As in embryos, tendon precursors of the flight and leg muscles originate in the epidermis and are first characterized by the expression of *sr* (Fernandes et al., 1996; Soler et al., 2004)*.* Although less commonly known, the initiation of *sr* expression in these contexts is dependent on local signaling factors that ensure the segmental subdivision of disc territories.

#### 2.2.1 Flight muscle attachment sites

In the wing imaginal disc, at the end of the second larval stage, *sr* is expressed in five clusters that are confined to the notum region, which gives rise to the thoracic dorsal body wall (Figure 1B). In the anterior part of the notum, there are four Sr+ domains: three in a lateral position and one in a medial position. The final cluster is localized in a more posterior position compared to the other Sr+ domains (Fernandes et al., 1996; Ghazi et al., 2003). Specification of tendon-like cells in wing imaginal discs is mediated by several regulatory interactions that includes the signaling pathways Wg, Decapentaplegic (Dpp), and Notch (Fernandes et al., 1996; Ghazi et al., 2003; Usui et al., 2004) and are summarized in Figure 1B. *Wg* is expressed in a narrow region of the presumptive notum between the large medial Sr+ domain and the three lateral domains, only partially covering them. Therefore, depending on their positioning, the different Sr+ domains receive different levels of Wg. The loss and gain of Wg signaling function suggest that a moderate level of Wg is required to initiate *sr* expression in some domains, however Wg can inhibit *sr* expression at a high level (Ghazi et al., 2003). In the same study, Ghazi et al. (2003) also investigated the role of Pannier (Pnr) and U-shaped (Ush), two transcription factors that mediate notum prepatterning. They showed that *pnr* expression overlaps with some Sr+ domains and that *sr* expression is altered in *pnr* mutant, suggesting that Pnr promotes the initial expression of *sr* in some domains. Conversely, Ush is known to antagonize Pnr function and act as a negative regulator of *sr* expression. The authors also showed that the Notch signaling pathway is a main activator of *sr* expression in all tendon precursors (Ghazi et al., 2003). A few additional factors regulate *sr* expression in tendon precursors of the flight muscle including *apterous* (*ap*), a LIM-homeodomain protein (Bourgouin et al., 1992; Ghazi et al., 2000) and members of the homeobox genes of the *Iroquois* family (*caupolican*, and *mirror*) that create the notum prepattern (Gomez-Skarmeta et al., 1996; Ikmi et al., 2008). Lastly, we can cite the conserved role, throughout the dipteran fly order, of the *achaete-scute* complex (*ac-sc*), which encodes transcription factors that regulate the development of sensory bristles (Cubas et al., 1991; Skeath and Carroll, 1991). Interestingly, *sr* and *ac-sc* are expressed in distinct domains of the notum and exhibit mutually antagonistic activities, leading to spatial segregation of tendon precursors and bristle precursors (Usui et al., 2004).

Altogether, these different functions demonstrate that the precise patterning of flight MAS is orchestrated by a complex regulatory network of prepattern genes and signaling pathways.

#### 2.2.2 Leg muscle attachment sites

In the legs, the main muscles consist of multiple fibers that are attached to a long internal tendon on one side and to a single MAS localized beneath the leg cuticle on the other side (Soler et al., 2004). Both types of tendon (long and cuticular) express *sr*, and the mechanisms that induce their expression are gradually being identified (Laddada et al., 2019; Laurichesse et al., 2021). From the third instar stage of larval development and the first hours of pupae metamorphosis, *sr* starts to be expressed in seven clusters of epithelial cells within the presumptive joints that will form the future connections between leg segments (Soler et al., 2004; Laddada et al., 2019) (Figure 1C). During the larval stage, leg segments are separated by cells forming concentric rings that fold to form the joints. This localized constriction of the disc epithelium is dependent on Notch pathway activation at segmental boundaries by its ligands Delta/Serrate (Celis et al., 1998; Bishop et al., 1999; Rauskolb and Irvine, 1999; Mishra et al., 2001; Rauskolb, 2001). Notch triggers the expression of the odd-skipped family of transcription factors that are responsible for inducing the invagination of the cell rings that form the joints (Hao et al., 2003; Ibeas and Bray, 2003). The clusters of Sr-positive cells appear at stereotypic positions within these odd+ rings. The initiation of *sr* expression is also Notch-dependent (Laddada et al., 2019). To explain the spatial restriction of *sr* expression within the rings of odd-expressing cells, it has been proposed that Notch signaling may cooperate with other local factors and signaling pathways that pattern the leg disc segmentation (Laddada et al., 2019). Among these factors are Wg and Dpp that determine the ventral and dorsal leg disc regions, respectively (Brook and Cohen, 1996; Lecuit and Cohen, 1997). Accordingly, the Wg pathway’s loss of function affects *sr* expression in specific tendon clusters (Laddada et al., 2019). Moreover, Wg and Dpp morphogens’ diffusion act in conjunction to regulate the expression of genes such as *Homothorax*, *Dachshund*, and *Distal-less* along the proximo-distal axis that pattern the segmental identities (Figure 1C); these genes may also influence the spatial segregation of *sr* expressing domains. Therefore, as in the wing disc and embryo, regionalization of *sr* expression in precise and stereotyped clusters can be attributed to a complex combination of permissive and negative factors that remain poorly understood.

## 3 Tendon cell differentiation and muscle-tendon interactions

### 3.1 Larval muscle guidance and terminal differentiation of tendon cells

#### 3.1.1 Muscle guidance

Larval abdominal body wall muscles exhibit a pattern of thirty muscles per hemisegment anchored to specific epithelial MAS that provide a well-defined orientation and positioning for each muscle. This highly stereotypical pattern is a particularly suitable model used to identify the cues that enable the correct connection between a given muscle and its specific attachment sites. The early phases of myogenesis, from mesoderm differentiation to myoblast fusion into syncytial myotubes, have been widely reviewed (Dobi et al., 2015; Schulman et al., 2015; Poovathumkadavil and Jagla, 2020). Once the myotubes have formed, filopodia grow from both extremities of the syncytial cell to search for and connect with their specific attachment sites (reviewed in Schnorrer and Dickson, 2004; Schweitzer et al., 2010). This indicates the presence of intrinsic (Carayon et al., 2020) and extrinsic mechanisms (i.e., released cues) that allow 1) the direction of myotube elongation and migration toward MAS, 2) the selection of the correct MAS, and 3) the end of their migration once they have reached MAS.

**FIGURE 2:**
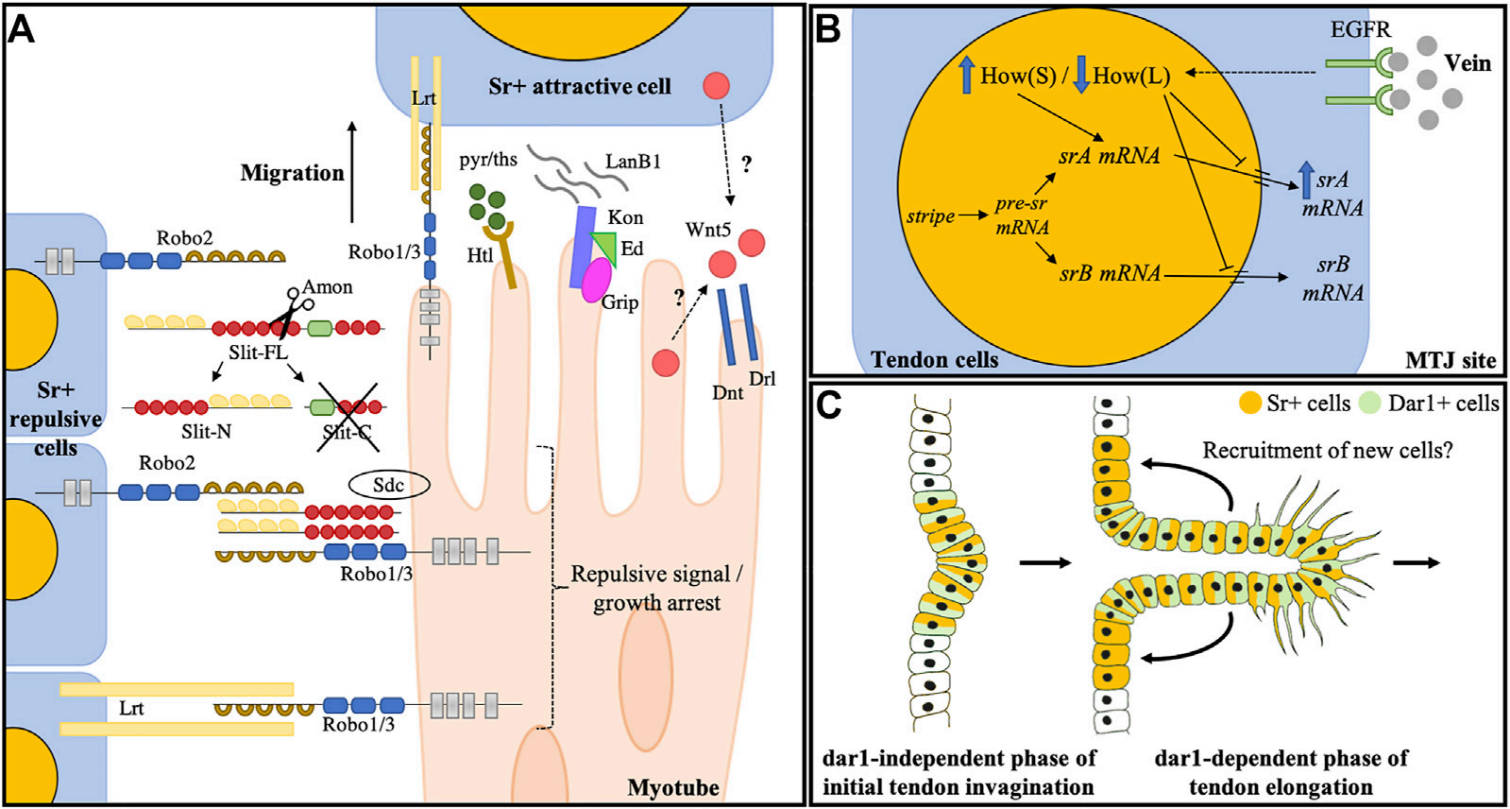
Larval muscle guidance during embryogenesis and tendon development of leg muscles. **(A)** Known molecular actors controlling myotube guidance/arrest toward tendon cells during embryogenesis. Slit is cleaved by Amontillado protease (Ordan and Volk, 2016) into a rapidly degraded Slit-C fragment and a Slit-N that remains tethered to the membrane of the expressing cell by binding to the robo2 receptor. Slit-N is interpreted as a short-range repulsive and/or arrest signal by approaching myotubes through its interaction with Robo1/3 and Syndecan co-receptors (Chanana et al., 2009). Later, it was proposed that Robo1/3 functions in the muscle arrest through its interaction with the LRT protein. Other molecules also impact muscle guidance: the complex Kon/Ed/Grip accumulates at the tip of some myotubes and interacts with LanB1to promote their migration. Wnt5 protein is secreted by both muscle and tendon cells and acts through Drl and Dnt transmembrane receptors to regulate muscle migratory behavior. FGF signaling is also an essential regulator of myotube guidance through the regulation of cytoskeletal regulatory proteins. Figure is adapted from Ordan and Volk (2016). **(B)** Terminal differentiation of tendon larval muscles. Myotubes reaching their site of attachment release the vein ligand. Vein accumulates at the MTJ and binds to its receptor (EGFR) at the tendon cell membrane. EGF pathway activation triggers a switch between How (L) and How (S) isoforms. Both How isoforms can bind *sr* mRNA, How (L) isoform represses the *sr* mRNA nuclear export and leads to its degradation and How (S) favors the splicing of the *srA* isoform, leading to upregulation of the srA protein that triggers tendon terminal differentiation. **(C)** Hypothetical model depicting dar1 function in long tendon development of leg. dar1-independent phase: In the first phase of long tendon morphogenesis, Notch and odd are responsible for epithelium folding (i.e., invagination). During this phase, Notch is also responsible for initiating the tendon progenitor commitment by inducing *stripe* expression in a few epithelial cells (Laddada et al., 2019). Stripe then induces *dar1* expression. dar1-dependent phase: During the second phase, dar1 regulates cytoskeleton remodeling and filopodia formation to promote collective cell migration and tendon elongation. In turn, the pulling mechanical forces may instigate the recruitment of new Sr-positive cells.

The Slit-robo ligand-receptor couple was the first identified signal that have been implicated in these mechanisms in some muscles. *Slit* encodes a Leucine-Rich Repeat protein that was first described as a dual protein with two opposing activities. In the early stages of development, robo expressing ventral myotubes migrate away from midline cells of the CNS that express Slit, preventing these specific ventral muscles from crossing the ventral midline. However, a few hours later, these same myotubes require robo to reach their attachment sites (Kramer et al., 2001). The apparent dual role of Slit/Robo signaling has been partially identified by studying Slit-expressing muscles (Ordan and Volk, 2016, 2015; Ordan et al., 2015). These studies demonstrate that a cleaved N-term fragment of the Slit secreted ligand remains bound to the membrane of cells that border the elongating muscles. This N-term fragment (Slit-N) provides a short-range repulsive signal that keeps migrating myotubes on their correct path and halts their elongation (Ordan et al., 2015) (Figure 2A). Interestingly, Slit-N tethering to the membrane is enabled by the robo2 receptor located at the surface of the tendon cell, whereas it binds to Robo1/3 expressed at the muscle membrane. Thus, Slit-N oligomers provide a short-range signal that mediates the link between tendon cells (Robo2) and myotubes (Robo1/3) (Ordan and Volk, 2015) (Figure 2A). Finally**,** once the muscle cell has reached its target attachment site, another leucine-rich repeat protein called Lrt, may interact with Robo receptors to mediate the arrest of muscle migration (Wayburn and Volk, 2009; Gilsohn and Volk, 2010). Lrt is a tendon-specific transmembrane protein and is positively regulated by sr (Wayburn and Volk, 2009). Accordingly, Lrt can physically interact with Robo receptors at the junction between the muscle and tendon and in *Lrt* mutant embryos, some muscles do not successfully attach and others do not arrest their migration behavior (Wayburn and Volk, 2009).

Although there is no clear evidence that each interaction between a given muscle and its attachment sites is systemically dependent on a dedicated tendon-specific signal, other components involved in myotube guidance and/or tendon cell selection have been characterized. For example, Wnt5 is specifically involved in the guidance and arrest of the lateral transverse muscles (Lahaye et al., 2012). The secreted Wnt5 protein interacts with Derailed (Drl) and Doughnut (Dnt), two receptor-tyrosine kinase-related proteins, to guide muscles to their corresponding attachment sites (Callahan et al., 1996; Lahaye et al., 2012). Another example is the protein complex Kon/Grip/Echinoid, where Kon-tiki (Kon) is a transmembrane protein required for the myofibril assembly that initiates the attachment of a subset of muscles (Schnorrer et al., 2007; Pérez-Moreno et al., 2014). Kon, with the intracellular protein Grip and the cell-surface protein Echinoid, forms a protein complex at the tip of the migrating myotube (Swan et al., 2004; Swan et al., 2006). *kon* and *grip* mutant embryos show a similar phenotype, where muscles are not properly guided to their attachment sites (Swan et al., 2004; Schnorrer et al., 2007; Pérez-Moreno et al., 2014). Moreover, Kon cooperates with the ECM protein Laminin LanB1 to regulate the migratory behavior of muscles in addition to facilitating the attachment itself (Pérez-Moreno et al., 2022). Lastly, FGF signaling has also been shown to regulate myotube morphogenesis and guidance (Yang et al., 2020; Yang et al., 2022). Although, the expression of *pyramus* (*pyr*) and *thisbe* (*ths*), two secreted ligands for the FGF receptor Heartless (Htl), is not restricted to specific tendon cells and is not directly expressed by tendon cells, their broad expression in the ectoderm appears to globally direct myotube leading edges toward their attachment sites (Yang et al., 2020).

#### 3.1.2 Tendon terminal differentiation

Several studies have contributed to an elegant model that explains how tendon cells enter terminal differentiation (previously reviewed in Schweitzer et al., 2010). Briefly: once a myotube has made contract with its attachment sites, it secretes vein, a ligand for the EGF receptor (Yarnitzky et al., 1997). Thanks to Short-stop (Shot), a spectraplakin family member, vein accumulates at the muscle-tendon junction and activates the EGF signaling pathway in the tendon cell (Yarnitzky et al., 1997; Strumpf and Volk, 1998). MAPKinase pathway activation in these cells initiates the switch between the long isoform of Held out wing [How (L)] and its short isoform How (S) (Nabel-Rosen et al., 2002, 1999). Both variants can bind *sr* mRNA at its 3′UTR, however How (L) inhibits its *sr* mRNA nuclear export leading to its degradation, whereas How (S) stabilizes it and favors *srA* isoform splicing leading to the upregulation of SrA protein (Nabel-Rosen et al., 1999; Volohonsky et al., 2007) (Figure 2B). Remarkably, Liu and Geisbrecht (2011) showed that muscle-specific expression of the canonical nuclear importin Moleskin (Msk) could play a cell non-autonomous role in the activation of the MAPKinase pathway in tendon cells, and therefore *srA* expression (Liu and Geisbrecht, 2011), although subsequent work from the same laboratory (Liu et al., 2013) suggests that this non-autonomous effect on MAPK activity is indirect and due to incomplete attachment or partial detachment of muscles. Still, these results support a model in which the late phase of tendon differentiation is muscle-dependent.

Thus, SrA characterizes mature tendon cells and regulates genes that promote the late differentiation program of these cells such as the bHLH transcription factor Delilah (Dei). Dei and Sr regulate the expression of cell adhesion molecules required for the establishment of the MTJ (Armand et al., 1994; Subramanian et al., 2007; Wayburn and Volk, 2009; Gilsohn and Volk, 2010; Egoz-Matia et al., 2011; Nachman et al., 2015).

### 3.2 Tendon development in the leg and adult thorax

As in embryogenesis, in leg and wing imaginal discs, the early cell fate decision of epithelial cells toward tendon lineage relies on the activation of *sr* (see above). However, only a few studies have examined the cellular and molecular mechanisms that control the later development of flight and leg muscle tendons. In the wing disc, Broad-complex transcription factors, upon 20-hydroxyecdysone regulation, are potential regulators of tendon cell terminal differentiation to ensure the correct attachment of flight muscles (Sandstrom et al., 1997; Sandstrom and Restifo, 1999). A RNAi screen performed in flight muscle tendon cells identified a few additional molecules that regulate the size of tendon cell clusters, including Tango1, an endoplasmic reticulum exit site protein involved in collagen secretion and that may also be implicated in MTJ formation (Tiwari et al., 2015).

In the leg disc, tendon cell clusters adopt a particular shape during metamorphosis. Each initial cluster of Sr+ cells undergoes a progressive invagination followed by a collective migration leading to the formation of a long polarized tube-like structure surrounding a central lumen (Soler et al., 2004; Laddada et al., 2019; Laurichesse et al., 2021) (Figure 2C). The initial invagination of leg tendon precursors appears to occur independent of sr activity. Instead, this process appears to be concomitant to the local folding of the leg epithelium at the junction between leg segments as tendon precursors are established among leg joint cells expressing *odd-skipped* genes (see above). However, the reduction in *sr* expression precludes the elongation of the tendon, suggesting that leg disc sr is required to trigger a differentiation program that allows the collective migration of these cells (Laddada et al., 2019). Interestingly, the expression of the Krüppel-like factor *dar1* is restricted to the appendicular long tendons and is not found in other tendon precursors that do not form long internal structures (e.g., tendons of the flight muscles or larval muscles) (Laurichesse et al., 2021). Moreover, Dar1 acts downstream of sr and participates in the cellular events required for tendon elongation such as the formation of actin-rich filopodia at the basal membrane of migrating cells (Figure 2C). Consequently, *dar1* knockdown leads to a shortening of the leg tendons and a reduction in the number of Sr+ cells though it is not required cell-autonomously to induce *sr* expression, suggesting that tendon elongation may indirectly participate in the recruitment of new Sr+ tendon cells from the epithelium (Laurichesse et al., 2021). Strikingly, throughout this process of long tendon development, subpopulations of leg muscle precursors are organized around each cluster of tendon precursors and remain firmly associated with them as the tendons elongate (Soler et al., 2004; Maqbool et al., 2006). Moreover, tendon development disruption affects the spatial distribution of these myoblasts (Soler et al., 2016). These observations suggest that tendon precursors could provide positional information to myoblasts and/or that myoblasts could contribute to tendon growth and elongation.

### 3.3 Setting up the myotendinous junction

Most of our knowledge about the setting up of the MTJ stems from studies of larval muscle attachment sites. However, a number of studies led on flight muscles suggest that, at least part of the proteins required for larval muscle MTJ assembly are also involved in the attachment of flight muscles to their respective tendons. Larval muscle-tendon adhesion is enabled through the interaction between numerous components of the ECM and the transmembrane receptor integrins. For instance, integrins mediate the link between the ECM and intracellular proteins (IAP: Integrin Associated Proteins) that connect to the actin cytoskeleton. These molecules were identified primarily by studying the MTJ of larval muscles, whose role was thoroughly reviewed by Maartens and Brown, 2015a. Here we focus mainly on the proteins that contribute to the ECM at the MTJ. The various integrin subunits are encoded by three genes: *multiple edematous wing* (*mew*/*αPS1*), *inflated* (*if*/*αPS2*) and *myospheroid* (*mys*/*βPS*). αPS and 1βPS subunits first heterodimerize at the tendon membrane, whereas the αPS2 and βPS link together slightly later on the muscle side to consolidate the MTJ (Maartens and Brown, 2015b). One of the main ECM proteins that interacts with the αPS2βPS integrin is the tendon-derived ECM protein called Thrombospondin (Tsp). Tsp accumulates at the MTJ and is essential to generate a functional MTJ; its absence leads to muscle disconnection from their attachment sites (Chanana et al., 2007; Subramanian et al., 2007). The Tsp-integrin interaction must be spatially and temporally regulated, as demonstrated by the examination of *slowdown* (*slow*) mutants. Slow is secreted by tendon cells and when mutated, Tsp accumulates prematurely at the growing end of the muscle leading to the formation of a weak muscle-tendon junction (Gilsohn and Volk, 2010). Moreover, Tsp localization is dependent on the proteoglycan protein Kon, which forms a protein complex with the αPS2βPS integrin (Pérez-Moreno et al., 2017). Thus, in addition to its role in muscle migration (Pérez-Moreno et al., 2022), Kon contributes to MTJ consolidation by recruiting αPS2βPS ligand, which would increase integrin-binding affinity to the ECM. These results also indicate that muscle guidance/migration and MTJ formation are two concomitant mechanisms rather than a step-by-step sequential process. It also demonstrates that the precise timing and amount of ECM components at the MTJ must be strictly controlled. As such, the muscle-derived ECM protein, Dystroglycan (Dg), appears to be post-transcriptionally regulated by *miRNA9a*. Whereas *Dg* is present in most ectodermal cells, its expression must be alleviated in the cells that differentiate into tendon cells*.* Upon *miRNA9a* deficiency, *Dg* is upregulated in the tendon cells resulting in aberrant muscle attachment. Furthermore, *Dg* overexpression in tendon cells affects ECM composition by modifying the level of βPS integrin subunits and laminin at the MTJ (Yatsenko and Shcherbata, 2014).

As mentioned above, other molecules have been found to participate in the MTJ and have been already listed by Maartens and Brown 2015a. However, others remain to be identified. For instance, a subset of proteins involved in hemolymph clotting, Fondue (Fon), the integrin-associated protein Tiggrin (Tig) and the Larval Serum Protein 1 Gamma (Lsp1), have been shown to accumulate at the junction between larval muscles and their respective attachment sites and to participate to the MAS architecture (Fogerty et al., 1994; Bunch et al., 1998; Green et al., 2016).

### 3.4 Tendon-cuticle interaction of flight muscles

Finally, on its opposing membrane (i.e., the apical membrane), the tendon cell must also bind to the cuticle (chitin-rich exoskeleton). As in vertebrate, tendons are at the intersection between smooth contractile tissue (muscle) and rigid tissue (skeleton or exoskeleton). During myogenesis, mechanical tension is required for myofibril sarcomere organization and formation (Lemke and Schnorrer, 2017; Luis and Schnorrer, 2021). However, this tension must be counterbalanced to avoid cuticle deformation. Using flight muscle tendons as a model, select studies have identified early molecular mechanisms that allow tendon cells to resist increases in tension during muscle maturation. At its apical membrane, tendon cells secrete Dumpy (Dpy) and Quasimodo (Qsm), two zona pellucida domain (ZPD) proteins (Wilkin et al., 2000; Chu and Hayashi, 2021). Dpy is a giant ECM protein that forms force-resistance filaments between tendon cells and the cuticle, whereas Qsm favors Dpy secretion and polymerization. Flies carrying some *dpy* or *qsm* mutated alleles display cuticle depressions at flight muscle attachment sites (Wilkin et al., 2000; Chu and Hayashi, 2021). This provides a strong cuticle anchor of tendon cells that creates mechanical resistance to muscle tension and prevents epithelium deformation (Chu and Hayashi, 2021). Within the tendon cell, the Chascon (Chas) adaptor protein is upregulated during tendon maturation. Interestingly, Chas localizes at the apical cortex of tendon cells and colocalizes with βPS-integrin at myotendinous junction, suggesting a role for Chas into the tendon cell to link the basal MTJ to the apical tendon-cuticle junction (Olguín et al., 2011). Chas acts through tendon-derived Jitterbug/Filamin (Jbug) and in cooperation with Myosin-II to respond to pulling forces that occur during muscle compaction (Olguín et al., 2011). It has been proposed that Chas and Jbug could contribute to the formation or behavior of the prominent arrays of F-actin fibers that connect the apical cortex of the tendon cell with the myotendinous junction (Alves-Silva et al., 2008; Olguín et al., 2011). Accordingly, Jbug and Myo-II form a complex with *Drosophila* Rho-kinase (DRok) to regulate elastic properties of the actin cytoskeleton to maintain the shape of the epithelium and polarity during the muscle compaction process (Olguín et al., 2011; Manieu et al., 2018). Interestingly, DRok also acts independent of Myo-II to maintain a stable connection between the tendons and muscle cells (Vega-Macaya et al., 2016).

## 4 Concluding remarks

Tendons are part of connective tissues precursors that are recognized as an important source of extrinsic cues that regulate skeletal muscle differentiation, growth, and patterning. Despite their critical importance, the understanding of connective tissue development lags behind that of other musculoskeletal system components in vertebrates. As described in this review, the *Drosophila* model shows a variety of muscle attachments that reflect the diversity of its somatic musculature. *Drosophila* tendons differ in several aspects: single cell or monolayer cells that ensure larval and flight muscle attachments are morphologically distinct from tube-shape tendons of the leg, different combinations of signaling pathways are required to specify the tendon cell fate in these different systems and specific transcription factors are differentially expressed in different tendons. Therefore, *Drosophila* are of great interest to uncover the developmental program of each tendon type. For instance, pioneer work has identified Sr/EGR as a central regulator of tendon cell fate, however more recent studies have identified a few new conserved factors that contribute to underlying specification and differentiation of particular tendons, thereby participating in tendon diversity. With the rapid development of high-throughput sequencing technology, comparative transcriptomic analysis would enhance our knowledge of the molecular mechanisms underlying tendon diversity during development. Lastly, tendon diversity may also result into differential composition of ECM between tendon types. In vertebrates, Collagen proteins are the major components of the MTJ (Subramanian and Schilling, 2015) whereas their importance in *Drosophila* muscle-tendon adhesion is less well-documented (Borchiellini et al., 1996; Urbano et al., 2009). It would be interesting to carry out a detailed analysis of MTJ components of the different tendons in order to determine whether some proteins of the ECM, such as Collagens, are specific to tendon types.

Finally, tendons and muscle connective tissues in general are associated with major clinical challenges, including tendon scarring and muscle dystrophies. A greater understanding of connective tissue cell differentiation and how they interconnect with muscles and the skeleton is critical to address mechanisms that contribute to pathologies.
